# The Natural History of Uterine Leiomyomas: Morphometric Concordance with Concepts of Interstitial Ischemia and Inanosis

**DOI:** 10.1155/2013/285103

**Published:** 2013-09-30

**Authors:** Gordon P. Flake, Alicia B. Moore, Norris Flagler, Benita Wicker, Natasha Clayton, Grace E. Kissling, Stanley J. Robboy, Darlene Dixon

**Affiliations:** ^1^Cellular and Molecular Pathology Branch, National Toxicology Program (NTP), National Institute of Environmental Health Sciences (NIEHS), National Institutes of Health (NIH), Department of Health and Human Services, Research Triangle Park, NC 27709, USA; ^2^Molecular Pathogenesis Group, National Toxicology Program Laboratory (NTPL), National Toxicology Program (NTP), National Institute of Environmental Health Sciences (NIEHS), National Institutes of Health (NIH), Department of Health and Human Services, Research Triangle Park, NC 27709, USA; ^3^Biostatistics Branch, Division of Intramural Research, National Toxicology Program (NTP), National Institute of Environmental Health Sciences (NIEHS), National Institutes of Health (NIH), Department of Health and Human Services, Research Triangle Park, NC 27709, USA; ^4^Department of Pathology, Duke University Medical Center, Durham, NC 27710, USA

## Abstract

Based upon our morphologic observations, we hypothesize and also provide morphometric evidence for the occurrence of progressive developmental changes in many uterine fibroids, which can be arbitrarily divided into 4 phases. These developmental phases are related to the ongoing production of extracellular collagenous matrix, which eventually exceeds the degree of angiogenesis, resulting in the progressive separation of myocytes from their blood supply and a condition of interstitial ischemia. The consequence of this process of slow ischemia with nutritional and oxygen deprivation is a progressive myocyte atrophy (or inanition), culminating in cell death, a process that we refer to as inanosis. The studies presented here provide quantitative and semiquantitative evidence to support the concept of the declining proliferative activity as the collagenous matrix increases and the microvascular density decreases.

## 1. Introduction

We hypothesize that many uterine leiomyomas (fibroids) undergo progressive obsolescence and eventual involution, largely as a result of the excessive elaboration of collagen into the interstitial matrix, thereby increasing the distance between tumor myocytes and their blood supply. Since the smooth muscle cells of fibroid blood vessels mirror the phenotypic transformational changes of the tumor myocytes, the blood vessels of fibroids also become progressively more fibrotic and hyalinized. Thus, tumor myocytes are subjected to a reduced supply of essential nutrients and oxygen as a consequence of both vascular and interstitial ischemia.

If the growth of fibroid tumors was solely dictated by those genetic and epigenetic changes that promote an increased proliferative rate, then the tumor myocytes should continue to proliferate and the tumor would continue to grow. On the other hand, if vascular and interstitial ischemia do develop within these tumors as the deposition of collagen continues, the proliferative capacity of myocytes would probably be diminished as the diffusion of nutrients and oxygen is impeded in both the fibrotic, thickened vessels and the fibrotic interstitium. In addition, if the rate of angiogenesis is not equivalent to or greater than the rate of fibrogenesis, the tumor myocytes would be subjected to the additional stress of an increased distance between myocytes and capillaries (reduced microvascular density).

With these concepts of excessive production and accumulation of collagen, reduced microvascular density, and combined vascular and interstitial ischemia in mind, we hypothesized that, in general, tumors composed of the highest percentage of collagenous matrix would have the lowest microvascular density and the lowest proliferative rate. The morphometric studies that we report herein offer support for this hypothesis.

## 2. Materials and Methods

### 2.1. Study Participants

Subjects were recruited from the George Washington University (GWU) Medical Center Obstetrics and Gynecology Department surgical rosters as part of the National Institute of Environmental Health Sciences (NIEHS) Uterine Fibroid Study. Informed consent was obtained, and the study was approved by the institutional review boards at the NIEHS and GWU. Details of patient recruitment, demographics, and collection of gross pathology data have been previously reported [1].

### 2.2. Developmental Phases of Fibroids

We arbitrarily categorized the fibroid tumors in a large fibroid study (NIEHS-Uterine Fibroid Study) into four phases on the basis of a microscopic estimation of the percentage of the tumor occupied by extracellular matrix in H&E-stained slides. All tumors in this study were therefore placed in 1 of the 4 phases on the basis of the following estimation:

 phase 1 = no, or insignificant, collagen matrix,
 phase 2 = <10% collagen,
 phase 3 = 10–50% collagen,
 phase 4 = >50% collagen.

### 2.3. Selection of Cases for Morphometric Study

Cases were selected at random from the previously phased fibroid tumors in the NIEHS Uterine Fibroid Study. Five fibroid tumors were selected from each of the 4 phases, for a total of 20 tumors. The only restrictions placed upon the selection of cases were as follows.

- Gross tumor size data must be available.
- There must be sufficient tissue in the paraffin block to be able to recut for special stains.
- The fibroid fragment must be large enough (>5 mm) to accurately evaluate the various parameters under study.
- There should be no cautery edge artifact or other significant histologic artifacts in the section.

### 2.4. Size of Tumors

Gross dimensions of the fibroid tumors were obtained from the fibroid worksheets used in the NIEHS Uterine Fibroid Study. Some tumors were only recorded as <2 cm or ≥2 cm.

### 2.5. Image Analysis of Collagen Content

Sections of fibroid tumors were cut at 5 *μ*m, placed on microscopic slides, and stained with Masson's trichrome stain. This stain provided an ideal contrast for our imaging purposes because the collagen is stained blue and the muscle is stained red. The Masson's trichrome-stained slides were then cleaned with an isopropanol solution and scanned with the Aperio Scanscope XT Scanner (Aperio Technologies, Inc., Vista, CA), a machine which uses line-scanning technology to capture high-resolution, seamless digital images of glass slides. After scanning, the slides were viewed with the Aperio Imagescope v. 11.1.2.752, a digital slide viewing and analysis program. Image analysis was performed using the Aperio colocalization algorithm. This algorithm calculates the contribution of multiple stains at every pixel location in the image. For the analysis of the Masson trichrome stains, the algorithm's parameters were set for the identification of all blue collagen staining present. The data output from the Aperio colocalization algorithm was expressed as the percentage of collagen present in each sample. This data was exported to an excel table and graphed.

### 2.6. Mitosis Counting

For the mitosis counts, as well as the PCNA and vascular counts, an Olympus BX50 microscope was used. The H&E-stained slides were scanned with the 20x objective until a mitosis was identified, and then the number of mitoses in 10 high-power (40x) fields was counted. Only structures consistent with the prometaphase, metaphase, anaphase, or telophase stages of mitosis in which hairlike chromatin fibers could be seen were counted. 

### 2.7. Immunohistochemistry

*PCNA*. Formalin-fixed, paraffin-embedded tissue sections (5 *μ*m thickness) were deparaffinized in xylene, rehydrated in ethanol and submerged in 3% hydrogen peroxide for 15 minutes. Antigen retrieval was achieved by microwave method with distilled water (power level 5 for two cycles of five minutes each). Tissue sections were incubated with primary antibody (Monoclonal mouse Anti-PCNA, Chemicon International, Inc., Temecula, CA) at a 1 : 1200 dilution (diluted equally in 1% nonfat dry milk and 1% bovine serum albumin (BSA)) for 30 minutes, followed by incubation with Goat anti-mouse IgM secondary antibody (Jackson ImmunoResearch Laboratories, Inc., West Grove, PA) at a 1 : 400 dilution (diluted in 1% BSA) for 30 minutes. Next, Streptavidin Peroxidase SS Label (BioGenex, San Ramon, CA) was applied to the sections for 30 minutes. All incubations were performed at room temperature in a moist chamber, and each step was followed by two rinses in 1X Wash Buffer (Dako, Carpinteria, CA). Visualization was performed with DAB chromagen for 6 minutes and counterstained in hematoxylin for 30 seconds. The area (or areas when necessary to count more than one) of the greatest staining with the PCNA antibody was chosen for the counts in each fibroid. All cells with nuclear staining were counted, regardless of the intensity. A total of 1000 cells, including both stained and unstained, were counted, using a 25 square (5 × 5) grid.

*Factor 8 (von Willebrand Factor)*. Formalin-fixed, paraffin-embedded tissue sections (5 *μ*m thickness) were deparaffinized in xylene and hydrated through a graded series of ethanol. The slides were then quenched in 3% hydrogen peroxide for 15 minutes at room temperature. Ready-to-use Carezyme Pepsin antigen retrieval solution (Biocare Medical) was added to the tissue slides and allowed to incubate for 5 minutes at 37°C. Afterwards, the tissues were blocked with normal serum from the Vectastain Rabbit Elite Kit (Vector Laboratories) for 20 minutes at room temperature. Rabbit anti-human Factor VIII Antibody (Biocare Medical) was applied to the tissues at 1 : 800 for one hour at room temperature. Normal rabbit serum (Jackson ImmunoResearch) was used instead of the primary antibody (at the same dilution as the primary antibody) for negative control staining. The secondary antibody and label complex reagents from the Vectastain Rabbit Elite Kit were both incubated on the tissue for 30 minutes at room temperature. The stain was visualized by using DAB chromagen (Dako) and hematoxylin counterstain. Lastly, the slides were dehydrated, cleared in xylene and coverslipped.

### 2.8. Microvascular Density (Factor 8 Counts)

Each tumor was scanned at low power (4x or 10x objective) to find the areas of most prominent vascular density with the Factor 8 stain. Vessels were counted with a 20x objective, and an eyepiece containing a 5 × 5 grid (each side = 750 *μ*m) to simplify the counting. Only structures that had the linear shape of vessels, whether with open or compressed lumina, or cross sectional profiles of vessels, were counted. Individual cells stained with the Factor 8 antibody were not counted. All vessels within the 5 × 5 grid, using the 20x objective, were counted in each of five different microscopic fields, and the five scores were then averaged to give an average microvessel density score for each fibroid.

The distance between vessels was also measured in some tumors, using the 20x objective and an ocular micrometer. This could only be accomplished with any accuracy in areas where several vessels had been cut in cross-section.

### 2.9. Statistical Analysis of Collagen Content, PCNA Proliferation Index, and Microvessel Density

Based upon the image analysis software evaluations of the mean percentage of collagen in the Masson trichrome-stained slides from each of the four phases, analysis of variance (ANOVA) and Fisher's least significant differences (LSD) multiple comparisons procedure were used to compare the percent of collagen among the four phases. Similar statistical procedures were used for the analysis of the PCNA proliferation indices and the Factor 8 microvessel density determinations.

### 2.10. Simulation Study of Myocyte to Vessel Distances

To supplement the analyses of the Factor 8 microvessel density studies, we used the Monte Carlo simulations to estimate the average and maximum distances between myocytes and blood vessels in phase 1 and phase 4 tumors. These simulations generated hypothetical 750 *μ*m × 750 *μ*m microscopic fields (the size of the eyepiece grid) in which blood vessels and myocytes were assumed to be uniformly distributed throughout areas not occupied by collagen. Although smooth muscle cells can vary considerably in size, for the purposes of these calculations, myocytes were assumed to be aligned in parallel and to be 100 *μ*m long and 10 *μ*m wide [2, 3]. It was further assumed that, in the absence of collagen, myocytes would fill the entire field such that there would be 750 *μ*m/100 *μ*m × 750 *μ*m/10 *μ*m = 7.5 × 75 = 562.5 myocytes per field. Blood vessels were assumed to be round with a diameter of 7 *μ*m. Mean numbers of blood vessels and percentages of collagen present in a 750 *μ*m × 750 *μ*m field were determined from the actual phase 1 and phase 4 tumors. Using these means, 500 hypothetical phase 1 fields and phase 4 fields were simulated, and for each field, the average distance and maximum distance were determined between the myocytes and their closest blood vessel. The likely ranges of average and maximum distances were estimated as the 2.5th percentile to the 97.5th percentile of the 500 simulated average or maximum distances, for each phase. Average distance and maximum distance were compared between phase 1 and phase 4 using *z*-statistics.

## 3. Results

### 3.1. Size of Tumors

Although exact measurements were not available for some tumors, all tumors were at least placed into a <2 cm or ≥2 cm category. On this basis, only 2 of the phase 1 tumors were >2 cm, while 3 of the phase 2 tumors were >2 cm, and all five of the tumors in both phase 3 and phase 4 were ≥2 cm (Table 1). Although the numbers are small, the data tend to support the concept that as fibroid tumors grow, most of them will accumulate extracellular matrix and that this continued deposition of collagen probably contributes, along with tumor cell proliferation, to the increasing size of the tumors. 

### 3.2. Phasing of Fibroids by Collagen Content

**Figure 1:**
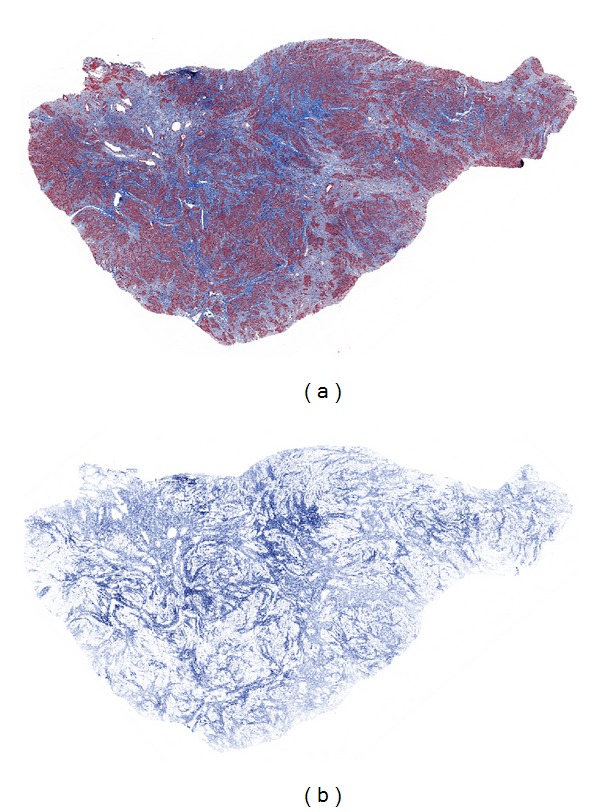
Image analysis of fibroid collagen content. By light microscopic examination of the H&E-stained section of this tumor, the collagen content was estimated to be more than 10% and less than 50% and thus to fall into the phase 3 category. The image on the left (a) shows the Masson's trichrome-stained section of this tumor, while that on the right (b) depicts the markup image of the same section in which only the blue-stained collagen has been colocalized, allowing for quantitation of the percent of collagen, which was found to be 38.4%.

**Figure 2:**
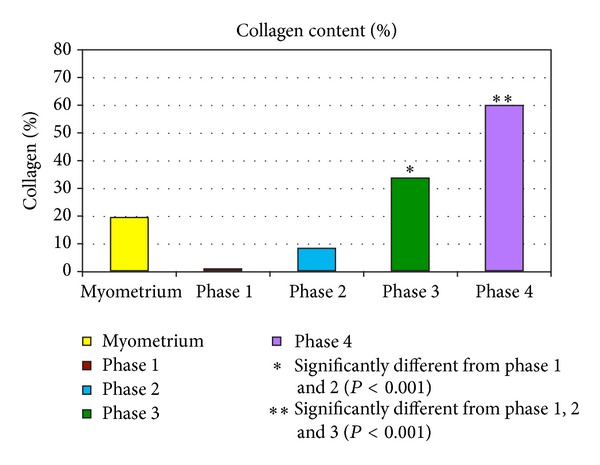
Mean percent collagen content of fibroid phases 1–4 by image analysis. The bar graph depicts the progressive accumulation of collagen in the transition from phase 1 to 4. There were 5 samples per phase group and the values represent the means. The myometrial bar is the mean value of 8 samples. *Significantly different from phases 1 and 2 (*P* < 0.001). **Significantly different from phases 1, 2 or 3 (*P* < 0.001).

In order to more accurately quantitate the percentage of collagen present and to evaluate the validity of the visual microscopic estimation, the microscopic sections of Masson's trichrome-stained slides of all 20 fibroids were scanned in the Aperio Scanscope and analyzed with Aperio Imagescope software (Figure 1). The individual tumor results within each phase are listed in Table 1, and the average percent of collagen for each phase is shown in Table 2. These data support the validity of visual estimation of the collagen content of fibroid tumors in H&E-stained slides. Almost all tumors within phases 2, 3, and 4 were found to have collagen components that fell within the estimated ranges of <10%, 10–50%, and >50%, respectively, and the collagen content of phase 1 tumors was minimal in each case (Table 1). In addition, the average values revealed broad differences between the phases, and there was no overlap in the collagen content of individual tumors between phases 2, 3, and 4. Finally, the computerized results demonstrate a progressive rise in the percentage of collagen from phase 1 to phase 4 (Figure 2), verifying the categorization of fibroids by visual estimation on H&E-stained slides.

Myometrial tissue was also available from 8 of the 20 patients in the study. The average collagen content of these 8 specimens was 19.4% (Figure 2), which exceeds the average collagen content of phase 1 and 2 tumors, and is lower than that of the phase 3 and 4 tumors. This intermediate collagen content of the myometrium is consistent with the notion that the early development of fibroids is marked primarily by myocyte proliferation, in contrast to the later phases (phases 3 and 4) in which there is excessive elaboration and accumulation of collagenous matrix. It is likely, however, that the collagen content of the myometrium is also variable and may be affected by a number of factors, such as the age of the patient, the reproductive history, and the location within the uterine wall.

Statistically, the percent of collagen differed significantly among the four phases (ANOVA *P* value < 0.0001, meaning that at least one phase was significantly different from the others). By Fisher's LSD, there was no significant difference in the percent of collagen between phases 1 and 2. Phases 1 and 2 were significantly lower than phase 3 and phase 4 (*P* < 0.001). Phase 3 was significantly lower than phase 4 (*P* < 0.001) (Figure 2). 

### 3.3. Proliferative Activity

Proliferative activity of fibroids has previously been shown to be increased in comparison to the myometrium [4]. As noted in Table 1, the tumors chosen for this study exhibited few mitotic figures, with only 1 tumor exhibiting more than 1 mitosis in 10 high-power fields. However, it is notable that none of the phase 4 tumors exhibited any mitotic activity. Of the 28 myometrial samples available from 8 of the 20 patients, no mitotic figures were identified. 

**Figure 3:**
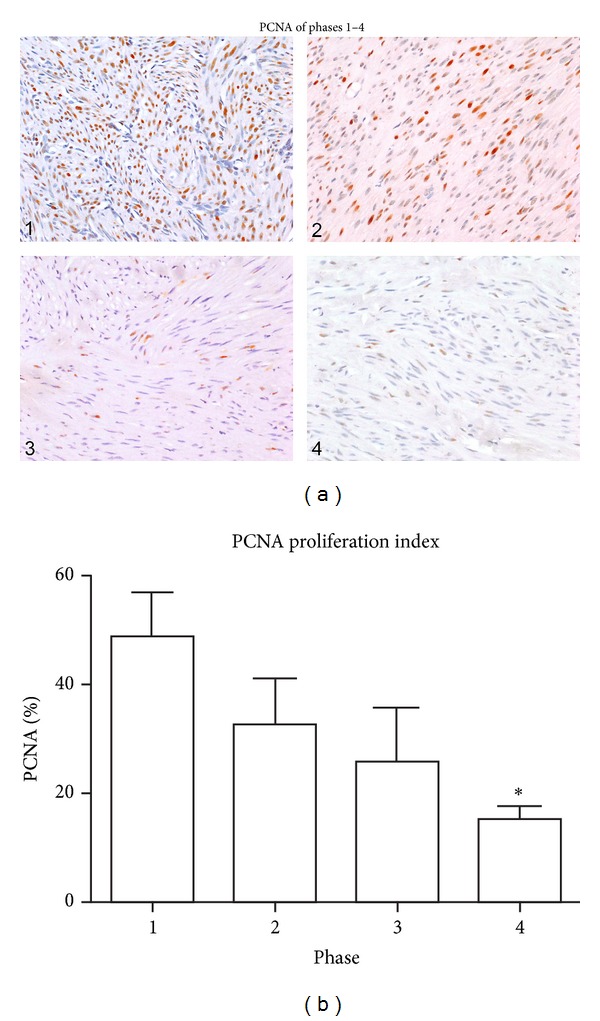
Fibroid phases: comparison of PCNA staining. (a) In this panel, a representative image of PCNA immunostaining from a fibroid in each of the four phases is shown. The percentage of PCNA positive nuclei in areas of maximum staining within the 4 fibroids in this panel was 59.1, 25.3, 19.1, and 15.5 for phases 1, 2, 3, and 4, respectively. A few PCNA positive nuclei are present in the phase 4 photo, but these are less intensely stained and thus less obvious than those in the other phases. All images were taken with the 20x objective. (b) Bar graph of Mean PCNAs from each phase. Although there was significant variation of PCNA scores within each phase of tumors in this study, as noted in Table 1, the mean scores of each phase declined progressively from phase 1 to phase 4. There were 5 samples per phase group and the values represent the mean ± SEM. *Significantly different from phase 1 (*P* < 0.01).

On the other hand, immunohistochemical nuclear staining for the proliferation marker, PCNA, revealed positive staining of some nuclei in all tumors. The percentage of positive staining cells (PCNA proliferation index) and the intensity of staining, varied from one area to another in each tumor. As noted in Table 1, the average percentage of nuclei staining with the PCNA antibody declined progressively from phase 1 to 4. Within the tumors of each phase, the number of PCNA positive nuclei was variable from one tumor to another, but notably the highest value in any of the phase 4 tumors (20.3%) was less than the lowest of the phase 1 tumors (24.3%), and the average PCNA value for the phase 1 tumors was more than 3 times that of the average value for the phase 4 tumors (48.6% versus 15.0%). Representative examples of PCNA staining are shown in Figure 3(a), and the average scores for each phase are depicted in the bar graph in Figure 3(b). 

The percent of PCNA labeling for phase 1 samples was significantly higher than that for phase 4 samples (*P* < 0.01). The percent of PCNA labeling for phase 2 and phase 3 samples was intermediate between phase 1 and phase 4 and did not differ significantly from either phase 1 or phase 4.

### 3.4. Microvessel Density (MVD)

**Figure 4:**
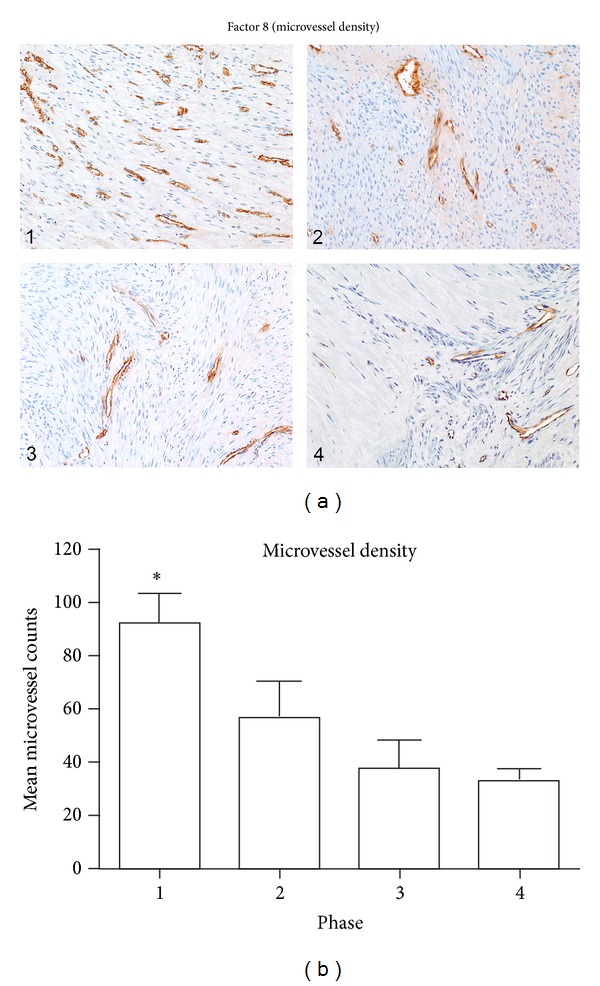
Fibroid phases: microvessel density. (a) In this panel, a representative photo of Factor 8 immunostaining from a fibroid in each of the 4 phases is shown. In some tumors the concentration of vessels was variable from one area to another, and within the fibroids of each phase there was a range of microvessel counts. However, the overall trend was one of decreasing vascularity with progression from phase 1 to 4. In phase 4 tumors, a prominent decrease in microvessel density was often noted within the hypocellular, hyalinized areas, as seen in the left half of the phase 4 tumor in (4). All images were taken with the 10x objective. (b) Bar graph of mean microvessel counts from each phase. Although the microvessel density varied among the individual tumors of each phase, the mean values declined from phase 1 to 4. There were 5 samples per phase group and the values represent the mean ± SEM. *Significantly different from phases 2, 3, and 4 (*P* < 0.05).

This parameter was assessed by immunohistochemical staining for Factor 8. The values listed in Table 1 represent the number of vessels counted with the 20x objective in a 0.5625 mm^2^ field (750 *μ*m × 750 *μ*m). The average values decline from a high of 92 in the phase 1 tumors to a low of 33 in the phase 4 tumors, a roughly threefold decline in MVD. Representative images from each phase are shown in Figure 4(a), and average microvessel counts for each phase are depicted in the bar graph in Figure 4(b). As noted in Table 1, there is a variation in the microvessel counts of individual fibroids within each phase, but the overall trend is one of decreasing MVD from phase 1 to 4. Variation in MVD was also apparent from one field to another of the same fibroid, particularly in phases 2, 3, and 4. For example, one of the phase 3 fibroids (2c) with a microvessel count of 8.8 displayed a higher level of vascularity (microvessel count of 19.2) in a section from another area of the same tumor (data not included in Table 1), and one of the phase 4 tumors (4d) with a microvessel count of 40.8 displayed other areas of reduced vascularity with counts of 23 and 24 (data not included in Table 1). In the Phase 4 tumors, the vascularity of hypocellular, hyalinized areas was sometimes equivalent to that of the residual cellular areas, while in other areas of hyalinization the MVD was considerably reduced as shown in Figures 4(a) (4), and 5. Since hyalinized areas with reduced vascularity were not included in the microvessel counts, it is likely that the overall MVD of the more fibrotic tumors, such as the phase 3 and 4 tumors, is even lower than measured; and further, that the difference in MVD between phase 1 and phase 4 tumors is probably greater than calculated since the hyalinized areas were not included. 

**Figure 5:**
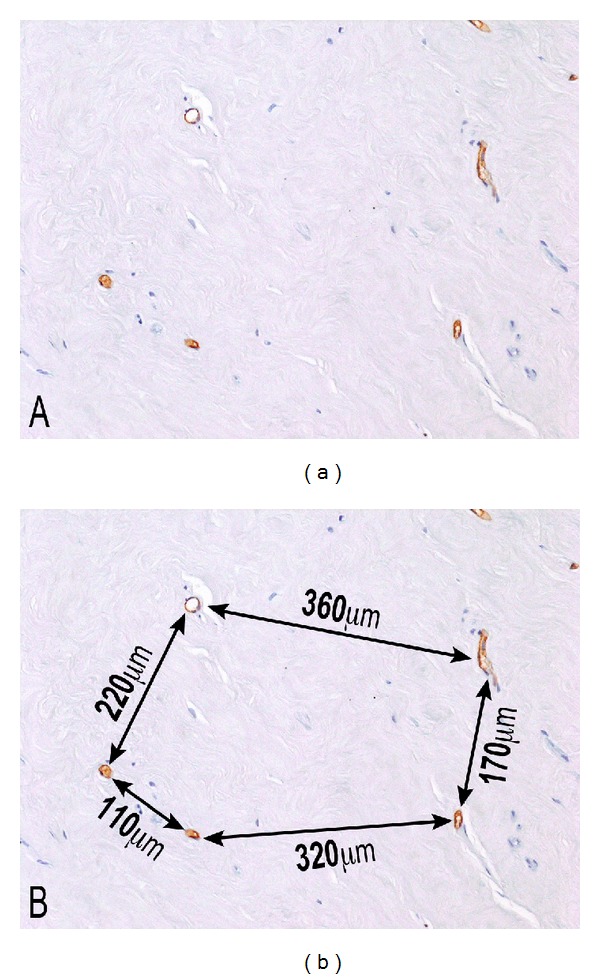
Phase 4 Fibroid. Hyalinized areas of fibroids, as in this phase 4 tumor, sometimes exhibit prominently reduced vascularity, in comparison to the adjacent areas of residual smooth muscle. Factor 8 immunostaining highlights the widely separated vessels in (a), and the distance between these vessels, measured with an ocular micrometer, is shown in (b). Note that the distances between adjacent vessels range from 110 *μ*m to 360 *μ*m, often exceeding the physiologic limits of cell survival, as evidenced by almost a total absence of cellularity within the intervening stroma. 10x original objective magnification.

**Figure 6:**
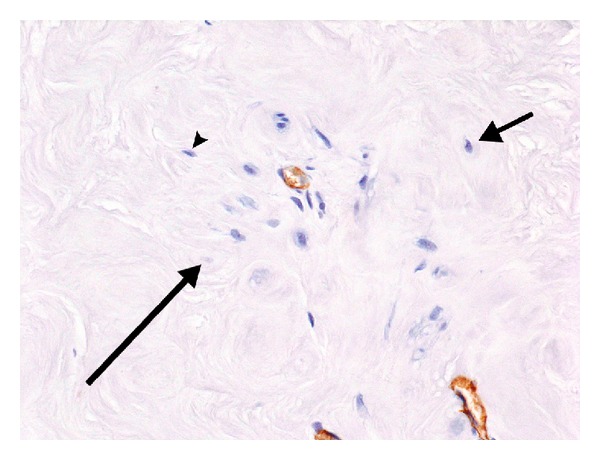
Phase 4 fibroid. Note that the only remaining viable cells in this field of a phase 4 fibroid are clustered around and between the two vessels. Most of these cells lie within 50–75 *μ*m of the 2 capillaries. The cell at 2 o'clock (short arrow) is located almost 100 *μ*m from the capillary in the center, and there are no viable cells beyond this point. The cell marked by an arrowhead is 50 *μ*m from the central vessel, and the apparent nuclear ghost (long arrow) is 70 *μ*m from the vessel. This suggests that in the setting of hyalinized fibroid stroma the maximum cell to vascular distance that is compatible with cell survival is in the range of 70–100 *μ*m, and that other factors such as the local density of the stroma may also play a role. 20x, anti-Factor 8 immunostain.

The distances between the vessels in a hyalinized area of a phase 4 tumor in Figure 5 ranged from 110 *μ*m to 360 *μ*m, with only a few surviving atrophic cells in the intervening hyalinized stroma, suggesting that the greater distances exceeded the physiologic limits of cell survival. In Figure 6, most of the cells lie within 50–75 *μ*m of the two vessels in the field. At the arrowhead, there is a lone surviving cell that is 50 *μ*m from the central capillary; at the short arrow, a single cell is noted that is approximately 100 *μ*m from the central vessel; and at the long arrow, there appears to be a nuclear ghost located 70 *μ*m from the central vessel. No visible cells are noted in the stroma beyond these points. Fields such as this suggest that the maximum cell to vessel distance compatible with cell survival in a hyalinized fibroid stroma is between 70 and 100 *μ*m. If a distance of 100 *μ*m between a cell and the nearest vessel is taken as the physical limit for cell survival [5], then the absence of cells, or the presence of nuclear ghosts, in some areas between 50 and 70 *μ*m lends credence to the concept that the regional density of the collagenous stroma in fibroid tumors probably offers an additional impediment to the free diffusion of oxygen and nutrients necessary for cell survival. 

The MVD, by Factor 8 staining, was significantly higher in phase 1 samples than in phases 2, 3 or 4 (*P* < 0.05). The MVD did not differ significantly among phases 2, 3 and 4 samples.

### 3.5. Simulation of Average and Maximum Myocyte to Vessel Distances

Simulation of the mean and maximum distances of myocytes from blood vessels in each phase is summarized in Table 3. Phase 1 tumors contained a mean of 92 blood vessels per 750 *μ*m × 750 *μ*m area and a mean collagen content of 0.83% (Table 1). Using this empirical data, the average distance of myocytes from blood vessels in Phase 1 tumors was estimated to be 34.9 *μ*m by Monte Carlo simulations (Table 3). The “likely range,” or the range within which one would find 95% of the averages, was 32.5 to 38.5 *μ*m. The maximum distance of myocytes from blood vessels in phase 1 tumors was estimated to be 141.4 *μ*m, with a likely range of 114.1 to 186.3 *μ*m.

Phase 4 tumors contained a mean of 33 blood vessels per 750 *μ*m × 750 *μ*m area and a mean collagen content of 59.99% (Table 1). Based on this empirical data, the average distance of myocytes from blood vessels in phase 4 tumors was estimated to be 58.0 *μ*m by Monte Carlo simulations, with a likely range of 50.5 to 68.4 *μ*m. The maximum distance of myocytes from blood vessels in phase 4 tumors was estimated to be 195.6 *μ*m, with a likely range of 144.2 to 283.4 *μ*m.

The differences between phase 1 and phase 4 tumors were statistically significant for both average (*P* < 0.001) and maximum (*P* < 0.001) distance of myocytes from blood vessels. 

## 4. Discussion

The results of these morphometric studies are supportive of the concepts and conclusions derived from our morphologic studies. The demonstration of an inverse relationship between the percentage of collagenous tumor matrix and the microvessel density is central to our thesis of interstitial ischemia, which, combined with vascular ischemia, leads to myocyte atrophy and eventual tumor involution.

Our findings also show the variation in collagen content, proliferative activity, and microvessel density within each of the fibroid phases 1–4. Overlap in values is also noted between individual tumors in adjacent phases (e.g., phases 3 and 4). However, these observations of variation within phases, and overlap between phases, do not invalidate the trends that are apparent by grouping fibroids into phases based on estimation of collagen content. What the variations and overlaps do indicate is the genetic and epigenetic heterogeneity of fibroids. Thus, some tumors may continue to proliferate and grow to a large size, with relatively little collagen production. Other tumors, in contrast, may produce abundant collagen early in development, resulting in interstitial ischemia with an associated reduced rate of proliferation and subsequent involution while still small in size. The majority of tumors, however, seem to exhibit a growth pattern between these two extremes, in which a more balanced progressive growth occurs as a result of both the proliferation of transformed myocytes and the production and deposition of collagen, until the tumors ultimately involute because of the accumulation of collagen in the stroma with secondary interstitial ischemia.

Cells ordinarily lie within 20–30 *μ*m of capillaries [6]. Proximity to vessels is essential for cell maintenance and survival since they are dependent upon diffusion of nutrients and oxygen from the blood stream. In our studies, the microvessel density of phase 1 tumors was 2.8 times the microvessel density of phase 4 tumors (92 versus 33 vessels/0.5625 mm^2^ field), while the percentage of collagen in phase 4 tumors was 72.2 times that of phase 1 tumors (59.99% versus 0.83%). Based upon these empirical data, the average distance of myocytes from blood vessels in phase 1 tumors was estimated, through simulations, to be 34.9 *μ*m, while the average distance in phase 4 tumors was estimated to be 58.0 *μ*m. These data reinforce our morphologic observations and our hypothesis that myocytes become progressively more separated from their blood supply as the tumors progress from Phase 1 to phase 4.

In addition to the increase in diffusion distance between myocytes and blood vessels, the density of both the fibrotic interstitial tissue and the vessel walls would be expected to increase the resistance to diffusion of nutrients and oxygen. The effective diffusion of molecules to the cells is dependent upon both the microvessel permeability and the tissue diffusion coefficient [7]. Although we are unaware of studies specifically investigating diffusion in fibrotic tissue, the reduction in ground substance within dense fibrotic tissue that is suggested histologically would probably be an additional impediment to the diffusion process.

The consequences of a reduction in the supply of nutrients and oxygen to cells are probably dependent upon both the degree of the reduction and the rapidity with which it occurs. While a sudden, marked decrease in blood flow might result in necrosis, a lesser degree of ischemia or hypoxia might induce an apoptotic reaction, and both types of cell death may be observed in fibroids. However, much of the cellular loss in fibroids appears to be related to a much slower process in which cells denied proper sustenance and oxygen because of the vascular and interstitial fibrosis undergo atrophy and injury. Ultimately, the fibrotic isolation of atrophic myocytes results in such severe deprivation of essential nutrients and oxygen that vital functions cannot be maintained and cell death occurs by inanition, or starvation, a process that we refer to as inanosis. Hypoxia is probably an important contributing factor to cell demise since it is believed that cells located more than 100 *μ*m from the vasculature develop anoxia, that is, complete oxygen deprivation [5]. The loss of myocytes by this more gradual process of atrophy and inanosis may also contribute to the extreme collagen to muscle ratios seen in the phase 4 tumors. 

Since the number of tumors examined immunohistochemically in this study was limited, additional studies are needed to confirm these results. Taken in conjunction with our morphologic studies, however, we feel that the data are supportive of the concepts of interstitial ischemia and inanosis.

We also recognize that each individual tumor can only be examined at one point in time and that certain assumptions are necessary to advance the idea of progression and ageing changes within individual fibroids because of this limitation. Thus, each tumor may pursue its own course with regard to rate of growth and the degree and rate of development of fibrosis, and cellular tumors with little fibrosis (phase-1-like) may be large, and fibrotic tumors (phase-4-like) may be small. However, the grouping of fibroids by the percentage of collagenous stroma to the percentage of residual muscle has allowed for the observation of trends that support the concepts of interstitial ischemia, atrophy, inanosis, and involution. The arbitrary classification of tumors into phases has been employed not for any implied clinical significance or utility, but rather for providing a framework for the investigation of the pathophysiologic concepts that we have proposed. Finally, it is not our intention to imply that the presence of involutional changes in fibroids causes them to disappear, or to undergo any predictable regression in size, or to pose any less of a clinical problem; in fact, the accumulation of extracellular collagenous matrix that is largely responsible for myocyte involution also appears to be a significant contributor to the increase in the size of fibroids which is a major cause of their clinical manifestations.

## Figures and Tables

**Table 1:** Summary of individual tumor data.

Fibroid	Size (cm)	Collagen^b^ (%)	Mitoses	PCNA (%)	Factor 8 (microvessel density)
Phase^a ^1					
1a	<2	1.14	1	24.3	92
2a	>2	0.17	0	54.7	79.2
3a	<2	1.86	1	69.9	113.0
4a	>2	0.61	0	35.2	56.6
5a	<2	0.37	0	59.1	119
Average	**2 > 2 cm**	**0.83%**	**0.4**	**48.6**	**92**

Phase 2					
1b	1.5 × 1.0 × 1.0	5.11	0	18.1	45.0
2b	>2	6.04	0	13.4	99.6
3b	>2	11.89	0	56.9	40.2
4b	4.0 × 3.0 × 2.5	3.33	1	25.3	25.4
5b	1.8 × 1.9 × 1.0	14.64	1	48.8	73.6
Average	**3 > 2 cm**	**8.20%**	**0.4**	**32.5**	**57**

Phase 3					
1c	>2	30.81	0	3.1	27.0
2c	4.0 × 5.0 × 3.0	36.55	0	8.2	8.8
3c	>2	33.36	0	19.1	27.6
4c	7.3 × 6.5	29.99	2	50.2	66.8
5c	3.5 × 3.0 × 2.0	38.41	0	47.6	56.4
Average	**5 > 2 cm**	**33.82%**	**0.4**	**25.6**	**40**

Phase 4					
1d	3.0 × 3.0	50.02	0	13.8	29.0
2d	6.0 × 5.0 × 4.0	72.65	0	19.1	43.6
3d	≥2.0	51.76	0	20.3	18.8
4d	14 × 7.5	67.42	0	6.3	40.8
5d	5 × 4.5	58.11	0	15.5	31.6
Average	**5 > 2 cm**	**59.99%**	**0.0**	**15.0**	**33**

^a^Phase was based upon light microscopic estimation of the percentage of collagen in the tumor in H&E-stained slides.

^b^Collagen (%) was determined by image analysis of Masson's trichrome-stained slides.

**Table 2:** Mean collagen content of phases 1–4.

Phase	H&E-stained slides collagen content (%)	Aperio colocalization % collagen (mean ± s.e.)
1^a^	No, or insignificant, collagen matrix	0.83 ± 0.30
2	<10%	8.20 ± 2.16
3	10–50%	33.82 ± 1.62
4	>50%	59.99 ± 4.39

^a^ *n* = 5 slide samples per phase group.

**Table 3:** Monte Carlo simulations of mean and maximum distances of myocytes from vessels.

	Mean distance, *μ*m	Maximum distance, *μ*m
Phase 1^a^	34.9 (32.5–38.5)	141.4 (114.1–186.3)
Phase 2	43.5 (39.8–49.1)	168.6 (132.2–235.1)
Phase 3	52.3 (46.1–60.0)	190.3 (143.6–263.2)
Phase 4	58.0 (50.5–68.4)*	195.6 (144.2–283.4)*

^a^ *n* = 5 slide samples per phase group.

*Statistically significant versus phase 1 (*P* < 0.001).

## References

[1] Moore AB, Flake GP, Swartz CD (2008). Association of race, age and body mass index with gross pathology of uterine fibroids. *Journal of Reproductive Medicine for the Obstetrician and Gynecologist*.

[2] Hendrickson MR, Kempson RL, Sternberg SS (1992). Uterus and fallopian tubes. *Histology for Pathologists*.

[3] Ross MH, Romrell LJ, Kaye GI (1995). Muscle Tissue. *Histology, Text and Atlas*.

[4] Dixon D, Flake GP, Moore AB (2002). Cell proliferation and apoptosis in human uterine leiomyomas and myometria. *Virchows Archiv*.

[5] Walshe TE, D’Amore PA (2008). The role of hypoxia in vascular injury and repair. *Annual Review of Pathology*.

[6] Hall JE (2000). The microcirculation. *Guyton and Hall Textbook of Medical Physiology*.

[7] Fu BM, Adamson RH, Curry FE (2005). Determination of microvessel permeability and tissue diffusion coefficient of solutes by laser scanning confocal microscopy. *Journal of Biomechanical Engineering*.

